# Concordance between muscle mass assessed by bioelectrical impedance analysis and by muscle ultrasound: a cross-sectional study in a cohort of patients on chronic hemodialysis

**DOI:** 10.1186/s12882-024-03487-0

**Published:** 2024-02-06

**Authors:** Eman Nagy, Emad Samaan, Mohamed El-Gamal, Muhammed Shamsuddin, Samar Tharwat

**Affiliations:** 1 Mansoura Nephrology and Dialysis Unit, Internal Medicine Department, Faculty of Medicine, Mansoura University, Mansoura, 35516 Egypt; 2 Forensic Medicine and Toxicology Department, Faculty of Medicine, Mansoura University, Mansoura, Egypt; 3 Medical Experimental Research Center (MERC), Faculty of Medicine, Mansoura University, Mansoura, Egypt; 4 Department of Biological sciences, Faculty of Science, New Mansoura University, New Mansoura, Egypt; 5 Rheumatology and Immunology Unit, Internal Medicine Department, Faculty of Medicine, Mansoura University, Mansoura, Egypt; 6 Department of Internal Medicine, Faculty of Medicine, Horus University, New Damietta, Egypt

**Keywords:** Hemodialysis, Muscle, Ultrasound, Sarcopenia, Chronic kidney disease

## Abstract

**Background:**

Sarcopenia is a common problem in hemodialysis (HD) patients, and it is diagnosed by low muscle mass, strength and/or low physical performance. Muscle ultrasound (US) is a non-invasive portable tool that might be used for assessment of muscle mass. The aim of the current study was to investigate the concordance between muscle US and bioelectrical impedance analysis (BIA) in diagnosis of sarcopenia in HD patients.

**Methods:**

This cross-sectional study included 41 HD patients. Sarcopenia was diagnosed according to the European Working Group on Sarcopenia in Older People (EWGSOP). The skeletal mass index was measured by BIA and the muscle strength was measured by handgrip strength. Muscle US was used to measure cross-sectional area (CSA) and thickness of quadriceps and biceps muscles.

**Results:**

The current study included 41 patients on HD (25 males), with a mean (SD) age of 44.18 (13.11) years and a median HD duration of 48 months. Sarcopenia was diagnosed in 58.5% of the patients. Patients with sarcopenia had significantly lower quadriceps muscle CSA than those without sarcopenia. The optimal cut-offs of quadriceps muscle CSA for both males and females for the diagnosis of sarcopenia were 2.96 and 2.92 cm^2^, respectively.

**Conclusion:**

Sarcopenia is prevalent among Egyptian HD patients. US on quadriceps muscle CSA could be used for diagnosis of sarcopenia in these patients.

## Introduction

Patients with chronic kidney disease (CKD) and end-stage kidney disease (ESKD) suffer from several metabolic abnormalities. These abnormalities, besides hemodialysis (HD), lead to a reduction of protein synthesis and an increase in protein catabolism, leading to a loss of muscle mass [1].

Irwin Rosenberg proposed the word “sarcopenia” in 1988 to describe the syndrome of muscle loss that affects the elderly [2]. Since then, consensus papers from geriatric societies have established and published a larger definition of sarcopenia which is “syndrome characterized by progressive and generalized loss of muscle mass and strength as well as poor physical performance with a risk of adverse outcomes including physical disability, poor quality of life and death” [3–7]. This syndrome may potentially be associated with the process of aging or other medical conditions, such as chronic kidney disease (CKD). The severity of the condition escalates in proportion to the decline in renal function [8–10].

Loss of muscle mass has been proposed as a useful factor for assessment of nutritional status, as it may aid to differentiate between patients with and without protein energy wasting (PEW). The skeletal muscle mass assessment, which is the chief component of lean body mass (LBM), may provide the most consistent data for diagnosing and monitoring PEW [11]. The existing gold standard imaging techniques such as computed tomography (CT) scan, magnetic resonance imaging (MRI) or dual energy X-ray absorptiometry (DEXA) are expensive and/or unsuitable for sequential routine use [12]. In addition, anthropometric or biochemical methods are poorly accurate, and they produce scarcely reproducible data for quantitative assessment [12].

Bioelectrical impedance analysis (BIA), which is a bedside technique, utilizes the various electrical properties of tissues and body fluids while applying an alternating low intensity electric current; the values of resistance and reactance allow an estimation of total body water and total body cell mass, especially helpful when multiple measurements are taken at the same time [13]. Hand grip strength (HGS) measures the strength of upper body muscle and has a good relation to ‘gold standard’ LBM measurements such as DEXA [14].

Muscle ultrasound (US) can be used for evaluation of muscle mass. Its major advantages, compared to other modalities, are represented by low cost, portability, and lack of radiation exposure [8]. US was utilized to evaluate muscle cross-sectional area (CSA) and its validity was established in patients with CKD who were not undergoing dialysis [15].

Due to the high prevalence of sarcopenia in HD patients and its hazardous effects on these patients, there is a need for a diagnostic method that is rapid, non-invasive, portable, and of low-cost. The gold-standard methods of assessing muscle mass such as CT, MRI, DEXA, and BIA are less available, expensive, and/or non-portable. To the best of our knowledge, there are only few studies that have investigated the clinical applicability of muscle US in the assessment of muscle mass [8, 16, 17], and in the diagnosis of sarcopenia in HD patients [18]. Thus, the aim of the current study was to explore the concordance between muscle mass assessed by BIA and by muscle US in maintenance HD patients.

## Materials and methods

### Patients and settings

This is a cross-sectional observational study that was conducted between August 2022 to February 2023. We included 41 maintenance HD patients who were dialyzed at Mansoura Nephrology and Dialysis Unit (MNDU). Adult patients who were more than 18 years old and who had been on HD for more than 6 months were included in the current study. Patients with physical impairment and significant comorbidities such as unstable hypertension (HTN), history of myocardial infarction, unstable angina, active liver disease, uncontrolled diabetes mellitus (DM), and advanced cerebral or peripheral vascular disease were excluded from the study. Those who had exhibited trauma to or amputation of upper or lower limbs or who were diagnosed with primary neuromuscular disease were also excluded. The study was carried out in accordance with the Declaration of Helsinki and was approved by the Institutional Research Board (IRB) of Mansoura University (Approval number: R.22.07.1768.R1). The study was explained to all the participants, and a signed informed written consent was obtained from them before the start of the study.

### Sample size calculation

Sample size was calculated by PASS software for Windows, version 11.0.8. PASS 11. NCSS, LLC, Kaysville, Utah, USA (www.ncss.com). Calculation relied upon a previous study by ***Battaglia et al.*** [19], who correlated quadriceps rectus femoris thickness by ultrasound with different body composition by BIA. A sample size of 38 achieves 91% power to detect a Pearson correlation of 0.600 using a two-sided hypothesis test with a significance level of 0.010. These results are based on 5000 Monte Carlo samples from the bivariate normal distribution under the alternative hypothesis.

### Sociodemographic and clinical data

The sociodemographic information of the patients, such as their age, gender, and marital status, was gathered. Furthermore, relevant clinical parameters, including the duration since starting HD therapy and the presence of diabetes mellitus (DM) or hypertension (HTN), were recorded.

### Blood sampling and laboratory tests

Blood samples were taken from the arteriovenous fistula before starting the first HD session of the week. An automated analyzer was used to perform routine laboratory tests, including blood hemoglobin, serum calcium, phosphorus, albumin, intact parathyroid hormone (iPTH), ferritin, and pre-HD urea. Blood sample for post-HD urea was drawn 15–20 s after the completion of the same HD session using the slow-flow method, then Kt/V was calculated by using the following equation.

Kt/V = [0.026 – Percent Reduction Urea (PRU)] – 0.460. PRU = [1 - (Post-HD urea ÷ Pre-HD urea)] [20].

### Muscle strength

Muscle strength was assessed using HGS as a quantitative metric. The hand grip dynamometer was utilized to test the grip strength after the HD session. The measurement was conducted while the patient was in a seated position, with the elbow flexed at a 90-degree angle and the forearm positioned in a neutral orientation. The measurement was conducted on three occasions in the non-fistula arm, and the highest recorded value was selected. The measurement was denoted in kilos [21].

### Muscle ultrasound

The ultrasound examinations were conducted by a rheumatologist who has a minimum of 8 years of experience in the domain of musculoskeletal ultrasonography (MSUS). The rheumatologist was blind to the clinical information regarding the study participants at the time of the US evaluation. The study utilized the EDAN U2 ultrasound equipment, manufactured in Shenzhen, China, which was equipped with a linear array transducer operating at a frequency range of 8 to 13.4 MHz. The frequency was adjusted to 13 MHz, and the sonographic parameters were optimized to achieve optimal imaging of the scanned muscles. Generous amounts of contact gel were applied to avoid compression of the muscles by the transducer.

Initially, the US was employed to measure the CSA and muscle thickness of the rectus femoris in the dominant quadriceps muscle according to previous studies [22, 23]. The patient was instructed to abstain from engaging in any strenuous physical activity for a period of 72 h. The placement of the transducer involved positioning its long axis in a perpendicular orientation to the dominant leg, specifically at a precise distance of 3/5 of the interval between the anterior superior iliac spine and the superior patellar border. The scanning depth was adjusted with care for the femur’s orientational capacity. In order to locate muscle septa prior to capturing images, the researchers conducted moderate contraction-relaxation maneuvers.

Subsequently, the transducer was positioned at the anatomical midpoint [24] between the elbow crease and the larger tubercle of the humeral head in order to assess the thickness and CSA of the biceps muscle at the dominant hand. The transducer was placed perpendicular to the long axis of the bices muscle with the arm muscles extended and relaxed.

**Fig. 1:**
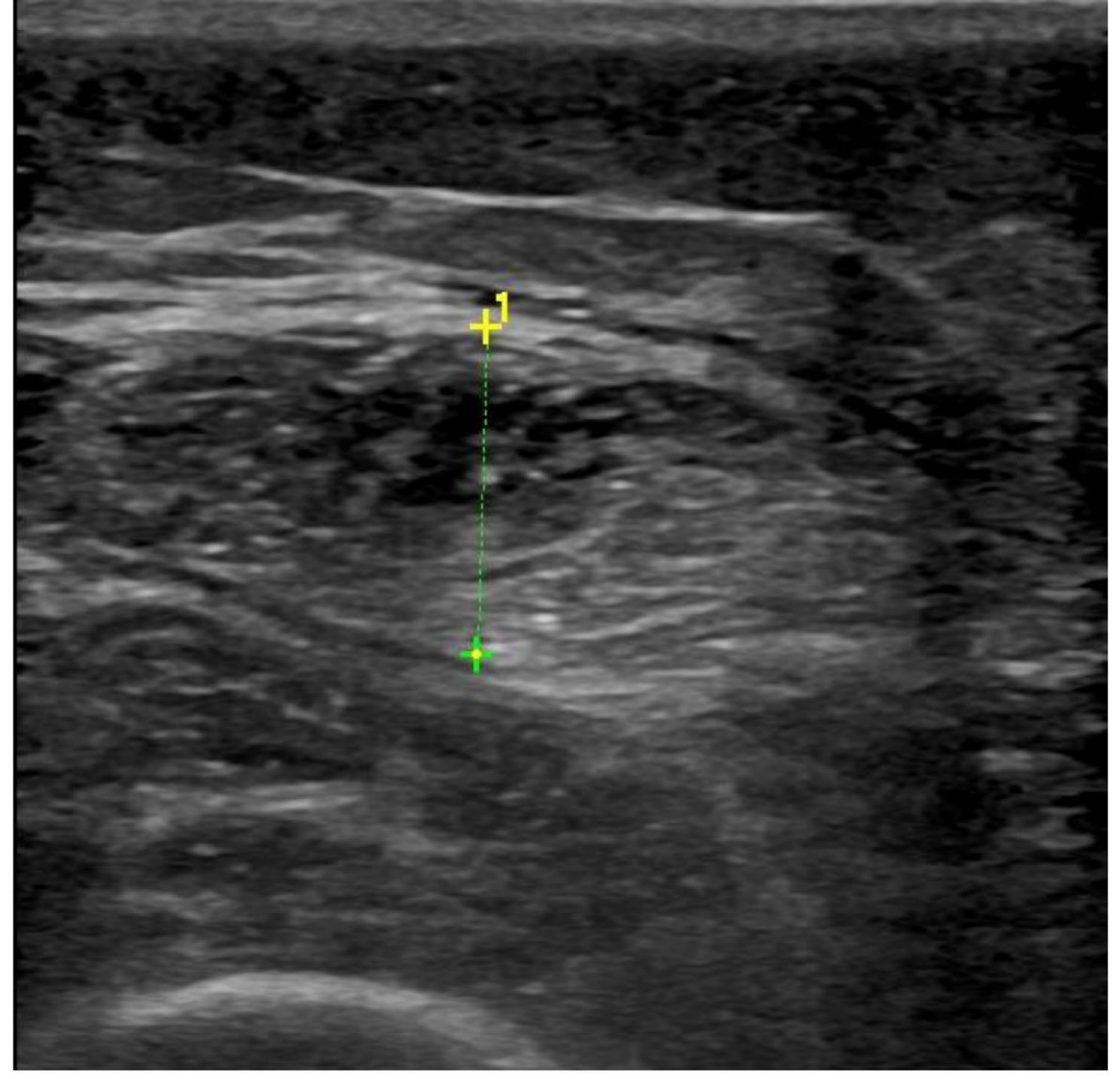
Rectus femoris muscle thickness. This figure depicts muscle thickness of rectus femoris perpendicular to its longitudinal axis in 35-year-old non sarcopenic male patients on HD for 4 years

**Fig. 2:**
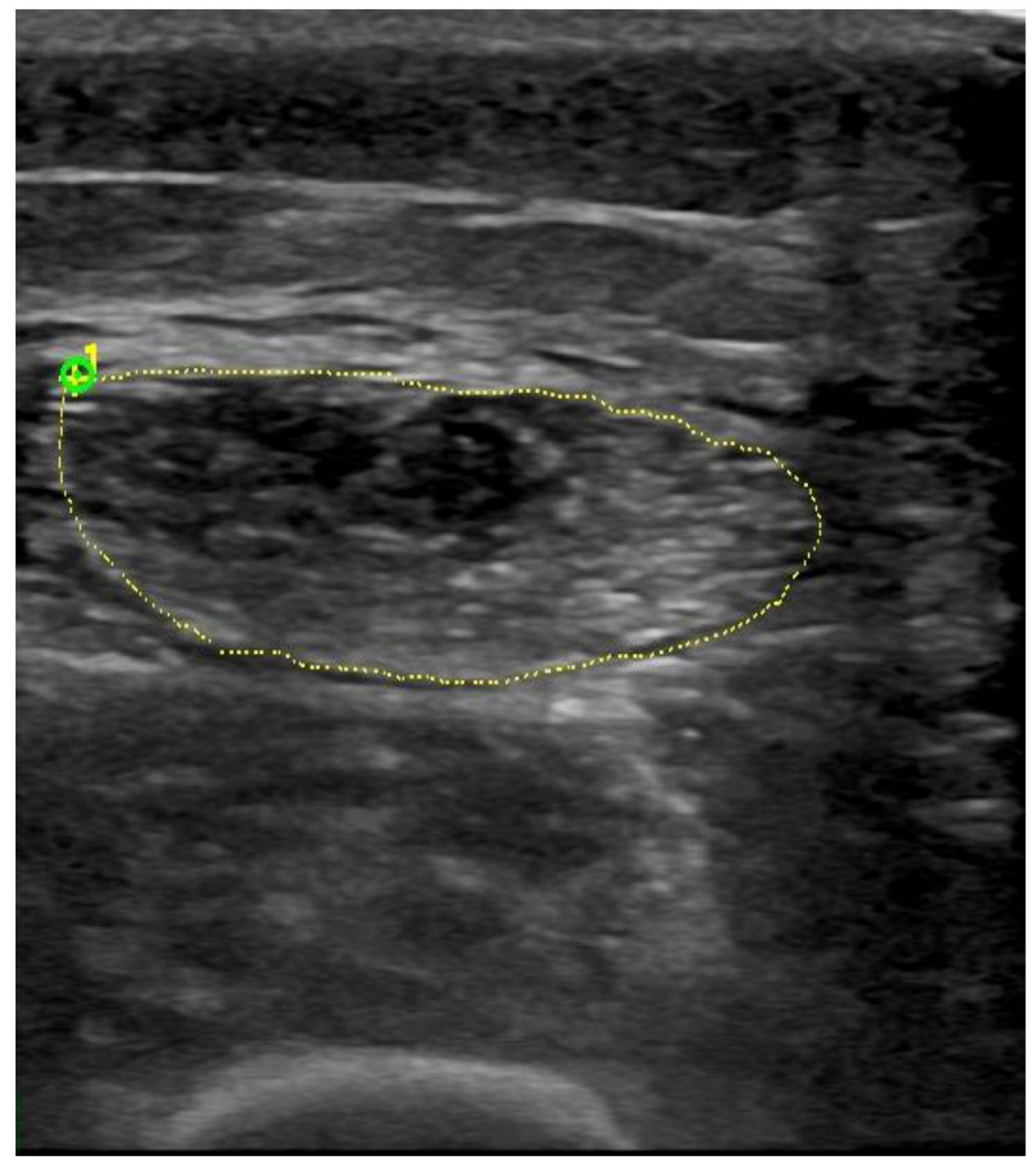
The imaging measurement of the cross-sectional area in the rectus femoris in the study patients

Images of the US were gathered for each individual and subsequently examined using proprietary software. On an image that had been frozen, a moving cursor followed the inner echogenic line of the rectus femoris and the biceps brachii muscles. Subsequently, the measurements of thickness and CSA were ascertained. Three consecutive readings were obtained for each measurement and subsequently averaged. Demonstrative cases are presented in Figs. 1 and 2.

### Body composition

Body composition was analyzed using the portable and precise InBody 270 body composition analyzer after the end of HD session. It provides accurate analysis for professionals on the go. It also delivers standard measurements like percent body fat, skeletal muscle mass, basal metabolic rate, and more. It quickly measures fat mass, muscle mass, and body water. It is also auto-calibrated, user-friendly, and non-invasive. The patient just stands on the device and hold the hand electrodes. Direct Segmental Multi-Frequency BIA technology measures body segments separately for an accurate analysis based on patient’s body. The following parameters were yielded from the InBody: Body mass index (BMI), total body water (%), body fat mass (kg), percent body fat (%), fat-free mass (kg), skeletal muscle mass (kg), and skeletal muscle index.

### Diagnosis of sarcopenia

According to the European Working Group on Sarcopenia in Older People (EWGSOP) [7], sarcopenia was diagnosed if the following criteria were met: (1) Low muscle mass: the skeletal muscle mass index (SMI) measured by BIA was less than 10.76 kg/m^2^ and 6.76 kg/m^2^ in males and females, respectively. (2) Low muscle strength: HGS in males and females was less than 30 kg and 20 kg, respectively. Based on these diagnostic criteria, the patients were divided into two groups: (1) patients with sarcopenia and (2) patients without sarcopenia.

### Statistical analysis

The collected data were coded, processed, and analyzed using Statistical Package for Social Science (SPSS) version 29 for personal computers. The parametric and non-parametric continuous data were expressed as mean ± SD and median (minimum-maximum), respectively. Categorical data was expressed as a number (percentage). The Shapiro-Wilk test was used to test for normality. The chi-square test was used to compare categorical variables. An independent t-test was used to compare parametric variables, while the Mann-Whitney test was used to compare non-parametric variables between groups. A receiver operating characteristic (ROC) curve was performed to allocate a cut-off point of quadriceps muscle cross-sectional area (CSA) to predict the presence of sarcopenia in the studied patients, and the cut-off point was chosen relying on the best possible specificity without sacrificing the sensitivity of choice. A *P* value less than 0.05 was considered significant.

## Results

The current study included 41 patients (25 (61%) males and 16 (39%) females), with a mean (SD) age of 44.18 (13.11) years and a median HD duration of 48 months. 78% of patients were married. Out of the total sample, a mere two individuals were diagnosed with DM, although a significantly larger proportion of 27 participants were found to have HTN. The patients were classified into two groups: patients with sarcopenia (24 patients, 58.5%) and patients without sarcopenia (17 patients, 41.5%). No statistically significant differences were found between the two groups in terms of age, gender, marital status, presence of DM and HTN, and the duration since starting HD. Furthermore, the laboratory data did not exhibit any statistically significant differences between the two groups, as illustrated in Table 1.

**Table 1:** Sociodemographic, clinical, and laboratory data of the studied patients (n = 41)

Variable	All patients (n = 41)	Patients without sarcopenia (n = 17)	Patients with sarcopenia (n = 24)	*P* value
**Sociodemographic data**:				
Age, years	44.18 ± 13.11	42.35 ± 8.57	45.47 ± 15.61	0.418*
Gender:				0.812***
Male	25(61%)	10(58.8%)	15(62.5%)	
Female	16(39%)	7(41.2%)	9(37.5%)	
Marital status:				0.056***
Married	32(78%)	16(94.1%)	16(66.7%)	
Non-married	9(22%)	1(5.9%)	8(33.3%)	
**Associated comorbidities**:				
DM	2(4.9%)	1(5.9%)	1(4.2%)	1***
HTN	27(65.9%)	13(76.5%)	14(58.3%)	0.228***
HD duration, months	48(6-170)	48(12–122)	54(6-170)	0.404**
**Laboratory data**:				
Blood hemoglobin, gm/dl	10.27 ± 1.44	10.61 ± 0.98	10.03 ± 1.67	0.176*
Serum albumin, gm/dl	3.99 ± 0.38	4.10 ± 0.39	3.91 ± 0.35	0.122*
Serum calcium, mg/dl	8.19 ± 0.82	8.46 ± 0.80	8 ± 0.79	0.084*
Serum phosphorus, mg/dl	5.32 ± 1.74	5 ± 1.75	5.54 ± 1.73	0.343*
iPTH, pg/mL	405(6.8–1559)	455(6.8–1559)	370(47.40–1467)	0.653**
Serum ferritin, ng/mL	282.6(5-1203)	374(32.6–1018)	221.5(5-1203)	0.296**
URR, %	60.88 ± 8.20	58.80 ± 7.92	62.61 ± 8.25	0.189*
Kt/V	1.16 ± 0.28	1.10 ± 0.24	1.22 ± 0.31	0.212*

The data were expressed as mean ± SD, median (minimum-maximum), or number (%), as appropriate.

*P value was calculated by independent t-test.

**P value was calculated by Mann-Whitney U test.

***P value was calculated by Chi-Square test.

Abbreviations: DM: diabetes mellitus, HD: hemodialysis, HTN: hypertension, iPTH: intact parathyroid hormone, URR: urea reduction ratio

Regarding body composition data, BMI, total body water, fat-free mass, skeletal muscle mass, and skeletal muscle index were significantly lower in the group of patients with sarcopenia. Patients with sarcopenia had significantly lower HGS than those without sarcopenia. Regarding muscle assessment by US, quadriceps muscle CSA was significantly lower in patients with sarcopenia than those without as shown in Table 2.

**Table 2:** Nutritional, BIA, and muscle US data of the studied patients

Variable	All patients (n = 41)	Patients without sarcopenia (n = 17)	Patients with sarcopenia (n = 24)	*P* value
Body weight, kg	79.20(47–158)	92(61.8-113.9)	76.4(47–158)	0.004**
Body mass index, kg/m^2^	29.30(18.50–54.70)	32.20(23.20–47.40)	27.25(18.50–54.70)	0.044**
**BIA**:				
Total body water, %	36.86 ± 7.40	40.24 ± 5.30	34.47 ± 7.83	0.012*
Body fat mass, kg	28.20(5.60–81)	35(10.20–65.50)	25.50(5.60–81)	0.199**
Percent body fat, %	37.16 ± 11.15	37.46 ± 11.52	36.94 ± 11.13	0.886*
Fat free mass, kg	50.15 ± 10.01	54.71 ± 7.26	46.91 ± 10.54	0.012*
Skeletal muscle mass, kg	27.71 ± 6.04	30.62 ± 4.45	25.65 ± 6.24	0.008*
Skeletal muscle index	7.56 ± 1.26	8.27 ± 0.75	7.06 ± 1.32	< 0.001*
**Muscle strength**:				
Hand grip strength, kg	19(4–55)	30(5–55)	12.5(4–25)	< 0.001**
**Muscle US**:				
Biceps muscle thickness, cm	1.79 ± 0.34	1.89 ± 0.29	1.70 ± 0.35	0.087*
Biceps muscle CSA, cm^2^	6.67(3.25–10.60)	6.97(4.97–10.60)	6.36(3.25–8.86)	0.229**
Quadriceps muscle thickness, cm	0.91(0.40–1.52)	0.96(0.60–1.5)	0.90(0.40–1.35)	0.219**
Quadriceps muscle CSA, cm^2^	2.82(1.36–5.50)	3.49(1.89–5.50)	2.23(1.36–4.05)	0.001**

The data were expressed as mean ± SD or median (minimum-maximum) as appropriate.

Bold means *P* value is significant.

*P value was calculated by independent t-test.

**P value was calculated by Mann-Whitney U test.

Abbreviations: CSA: cross-sectional area

**Fig. 3:**
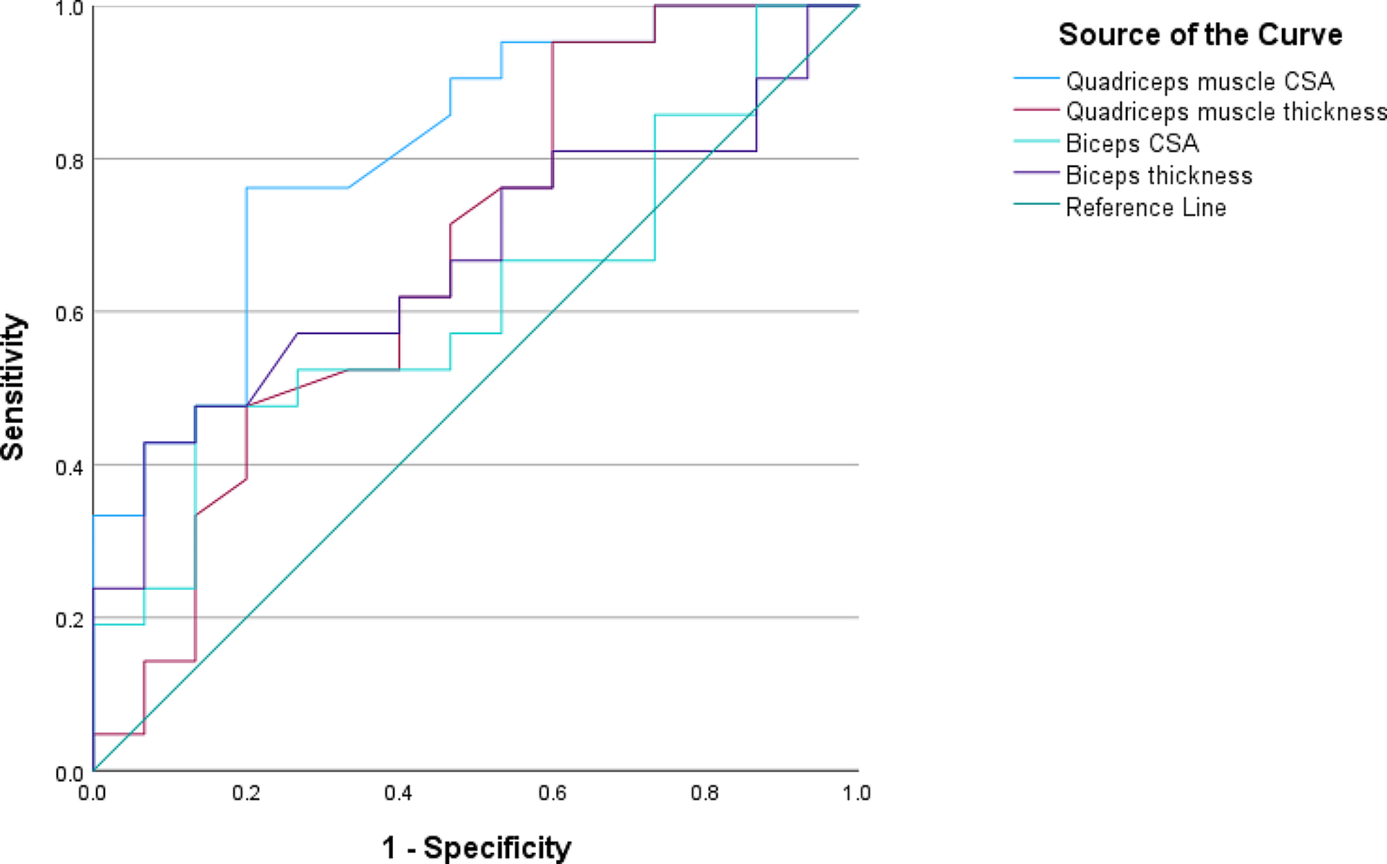
ROC curve of different muscle parameters assessed by ultrasound as predictors of sarcopenia. Abbreviations: CSA, cross-sectional area

**Fig. 4:**
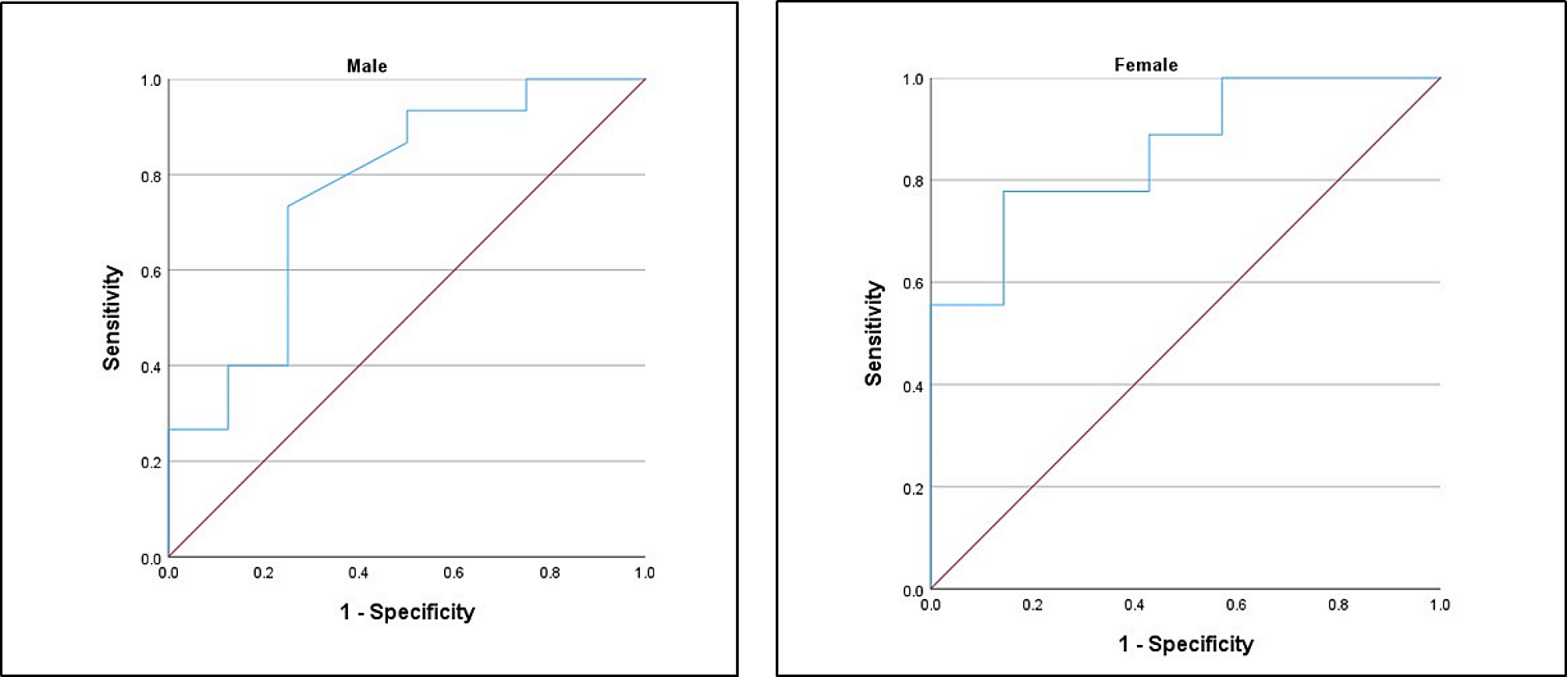
ROC curve of quadriceps muscle cross-sectional area assessed by ultrasound in both males and females as a predictor of sarcopenia

By constructing ROC analysis to test US measurement of the biceps and quadriceps femoris for detection of sarcopenia. Only the ROC curve of the quadriceps muscle CSA was statistically significant (*P* = 0.001) with an area under the curve (AUC) for the diagnosis of sarcopenia of 0.741. Using a quadriceps muscle CSA optimal cut-off value of 2.92 for all patients, as determined by the Youden index, the corresponding sensitivity and specificity for detection of sarcopenia were 75% and 73.3%, respectively (Table 3; Fig. 3). The optimal cut-offs of quadriceps muscle CSA for both males and females for the diagnosis of sarcopenia were 2.96 and 2.92 cm^2^, respectively (Table 3; Fig. 4).

**Table 3:** ROC curve of different muscle parameters assessed by ultrasound as predictors of sarcopenia

	AUC	*P* value	Cut-off	Sensitivity	Specificity	PPV	NPV
Quadriceps muscle CSA:							
All patients	0.807	0.001	2.92	75%	73.3%	81.8%	64.7
Male	0.767	0.039	2.96	73.3%	75%	84.6%	60%
Female	0.857	0.017	2.92	77.8%	85.7%	87.5%	75%
Quadriceps muscle thickness	0.624	0.199	1.20	87.5%	33.3%	67.7%	62.5%
Biceps CSA	0.641	0.138	5.98	47.6%	88.2%	83.3%	57.7%
Biceps thickness	0.667	0.081	1.52	42.9%	88.2%	81.8%	55.6%

Abbreviations: AUC: area under the curve, CSA: cross-sectional area, NPV: negative predictive value. PPV: positive predictive value, ROC: receiver operating characteristic

## Discussion

Sarcopenia is prevalent among HD patients, and it is associated with different health hazards [25, 26]. Its diagnosis depends on an assessment of muscle mass and strength in addition to physical performance [7]. Muscle mass is assessed by several tools including radiological and non-radiological methods [27]. The aim of the current study was to assess the potential of quadriceps muscle thickness and CSA as well as biceps muscle thickness and CSA measured by US as indicators for the presence of sarcopenia in HD patients. Sarcopenia was detected in 58.5% of the studied patients. From all the US measures, only quadriceps muscle CSA was a significant predictor for the diagnosis of sarcopenia in the HD patients, with a cut-off value for the diagnosis of sarcopenia of 2.92 cm^2^ in all studied patients. In addition, its optimal cut-offs for both males and females for the diagnosis of sarcopenia were 2.96 and 2.92 cm^2^, respectively.

In the current study, more than half of the patients (58.5%) were diagnosed with sarcopenia. This percentage is higher than reported by Sánchez-Tocino et al. [28] who studied 60 HD patients older than 75 years old and concluded that 37–40% of the patients had confirmed sarcopenia. In addition, this percentage was much higher than that of the study by Abdala et al. [29] who found that 16% of studied patients had sarcopenia. Also, our reported prevalence is slightly higher than that stated by Fu et al. [30] who found that 51.3% of HD patients had confirmed sarcopenia. Furthermore, Anderson et al. found a lower percentage of HD patients, compared to the current results, to have sarcopenia [31]. A recent systematic review and meta-analysis, which included 30 studies, reported a wide variation in the prevalence of sarcopenia from 4 to 68% among patients on dialysis [32]. This wide difference in the prevalence of sarcopenia among patients on HD might be due to different patient age groups, socioeconomic characteristics, and locations, in addition to using different diagnostic criteria for the diagnosis of sarcopenia. In addition, patients’ comorbidities might be attributed to this difference. In the current study, patients with uncontrolled DM were excluded from the study. Only patients with uncomplicated DM were recruited. That was because DM may accelerate the occurrence of sarcopenia. Previous studies reported a strong association between DM and sarcopenia [33].

In the current study, the mean total body water was 36%. This result is consistent with other studies [34–36]. In addition, total body water was significantly lower in patients with sarcopenia. There was a proposed relationship between intracellular water and muscle mass that swelling of intracellular compartments might induce glycogen synthesis and intracellular water depletion might result in protein degradation [37, 38]. Thus, lower total body water, including intracellular water, might be related to increased risk of sarcopenia. As the diagnosis of sarcopenia was dependent on assessment of muscle mass, skeletal muscle mass was significantly higher in patients with sarcopenia than those without.

Muscle mass can be assessed by several tools, such as anthropometry, BIA, DEXA, CT, and MRI. Anthropometric measurement represented by mid-arm muscle circumference (MAMC), which is derived from mid-arm circumference and triceps skin fold thickness, might be used for evaluation of muscle mass in HD patients [39]. These anthropometric measurements are easy, cheap, applicable, non-invasive, and can be done by bedside examination; however, they have some limitations, such as low reproducibility and reliability, in addition to being affected by other factors such as the hydration status of patients [40]. BIA assesses body composition according to the body resistance to the passage of an alternating electrical current [41]. BIA is non-invasive and easy to perform, in addition to being a bedside technique. However, it is not available at all HD centers, and in certain circumstances, it can be affected by the hydration status of HD patients [42, 43].

Regarding imaging techniques, DEXA can identify different components of body weight in addition to providing both segmental and whole-body parameters of body composition. In addition, DEXA is suggested as the reference method to evaluate muscle mass, particularly appendicular skeletal mass, and for the diagnosis of sarcopenia in the most recent Sarcopenia, Cachexia, and Wasting Disorders position paper [44]. However, DEXA has several limitations, such as being affected by the patients’ hydration status, cost, radiation exposure, the need for an experienced radiology operator, and being non-portable [41]. There are other imaging techniques used for muscle evaluation, such as CT and MRI. They are non-invasive and they are considered the gold standard for evaluation of muscle mass [3, 7]. CT is not influenced by hydration status. It can assess quadriceps muscle CSA and volume in addition to muscle density [45, 46]. On the other hand, CT is expensive, non-portable, exposing patients to ionizing radiation and not available in all HD centers. MRI can be used for assessment of psoas and quadriceps muscle CSA [47]; however, it is expensive, not portable, and not available at all HD centers in addition to needing specialized personnels.

Due to the limitations of the aforementioned methods for evaluating muscle mass, a search for another technique that overcomes these limitations is required. Muscle US is safe, portable, and not expensive [48]. In addition, there is no need to expose patients to ionizing radiation, and it provides real-time visualization of the muscles. Through muscle echogenicity, it can give information about the existence of inflammation, fibrosis, and adipose infiltration [49]. The validity and reliability of muscle US have been well supported in the renal setting for the evaluation of quadriceps muscle CSA and thickness. Souza et al. [50] have concluded that in patients with pre-dialysis CKD, rectus femoris CSA evaluation with US has been shown to be a viable and reliable procedure. In another study on 34 critically ill patients with KDIGO stage 3 acute kidney injury (AKI), muscle US of the rectus femoris and vastus intermedius was proven to be a simple, accurate, and non-invasive method for assessing quantitative changes in quadriceps femoris muscle [17]. Another study on patients with AKI by Sabatino et al. [51] revealed that quadriceps muscle thickness evaluated by US is consistent with CT measures.

Regarding HD patients, there is one study that tested the applicability of quadriceps muscle CSA assessed by US in evaluating muscle mass and diagnosing sarcopenia. Matsuzawa et al. [18] studied 58 HD patients and concluded that US of the rectus femoris CSA identified patients with high risk of sarcopenia and could be a valid diagnostic technique in these patients. In the current study, quadriceps muscle CSA is a significant predictor of sarcopenia in HD patients and could be a valid diagnostic tool for sarcopenia in these patients. In addition, the cut-off for quadriceps muscle CSA discriminating those with sarcopenia based on the Youden index was 2.96 cm^2^ for males and 2.92 cm^2^ for females, respectively. These cut-offs were higher than reported by Matsuzawa et al. [18], who found cut points for the diagnosis of muscle wasting to be 1.88 and 1.43 cm^2^ in male and female HD patients, respectively.

The limitations of the current study include relatively small sample size with different proportions of males and females and the nature of single center study. Additionally, patients with uncomplicated DM were included in the study, that was because there were a good percentage of HD patients with DM. Thus, not all patients with DM could not be excluded. In addition, the cross-sectional design of the study is one of the limitations. Larger studies are needed to set up a cut-off value for quadriceps muscle CSA for diagnosis of sarcopenia in HD patients. Furthermore, studies with longitudinal design are required to identify the fluctuations of muscle mass and to investigate the potential factors that might afflict the muscle mass of HD patients.

## Conclusion

Sarcopenia is prevalent among Egyptian HD patients. US on quadriceps muscle CSA could be used for diagnosis of sarcopenia in these patients. The cut offs for diagnosis of sarcopenia in these patients were 2.96 and 2.92 cm^2^ in males and females, respectively.

## Data Availability

The datasets generated and analyzed during the current study are not publicly available due to local university policy but are available from the corresponding author on reasonable request.
